# Chemical Fate of Ascorbic Acid in Wheat Flour Extract: Impact of Dissolved Molecular Oxygen (O_2_), Metal Ions, Wheat Endogenous Enzymes and Glutathione (GSH)

**DOI:** 10.3390/molecules30122582

**Published:** 2025-06-13

**Authors:** Alice S. Beghin, Sambhu Radhakrishnan, Nand Ooms, C. Vinod Chandran, Karel Duerinckx, Bram Pareyt, Kristof Brijs, Jan A. Delcour, Eric Breynaert

**Affiliations:** 1 Laboratory of Food Chemistry and Biochemistry and Leuven Food Science and Nutrition Research Centre (LFoRCe), KU Leuven, Kasteelpark Arenberg 20, B-3001 Heverlee, Belgium; 2 Center for Surface Chemistry and Catalysis—Characterization and Application Team (COK-KAT), KU Leuven, B-3001 Heverlee, Belgium; 3 NMR/X-Ray Platform for Convergence Research (NMRCoRe), KU Leuven, B-3001 Heverlee, Belgium; 4 Puratos NV, Industrialaan 25, B-1702 Groot-Bijgaarden, Belgium

**Keywords:** ascorbic acid, wheat breadmaking, chemical fate

## Abstract

Ascorbic acid (AH_2_) is a commonly used additive in food products. In wheat breadmaking, it is, for example, added to flour for its dough strengthening and bread volume-enhancing effects. While these bread property-enhancing effects are well known, the final chemical fate of AH_2_ in breadmaking applications remains nearly undocumented. This study tries to shed light on the chemical fate of AH_2_ in wheat breadmaking by investigating the chemical and enzymatic conversion of AH_2_ and its reaction products using ^13^C NMR spectroscopy in combination with AH_2_ labelled with ^13^C on the C_3_ carbon. Following the chemical conversion of AH_2_ as function of time, in ultra-pure water, tap water, and wheat flour extracts, in the presence and absence of dissolved O_2_ and glutathione (GSH), the specific impact of the presence of trace metal ions, dissolved oxygen and endogenous GSH on the oxidation of AH_2_ could be elucidated.

## 1. Introduction

Ascorbic acid (AH_2_), also known as vitamin C, plays a crucial role in human health due to its involvement in various physiological processes [1,2,3,4]. Its importance was first recognized in 1932 by Nobel Laureate, Albert Szent-Györgyi, who discovered this water-soluble micronutrient. Since then, ascorbic acid has been the subject of extensive research, initially for scurvy prevention and later also for its immune system boosting properties, its role in antioxidant defense mechanisms and collagen synthesis-enhancing effects. Besides being a micronutrient, AH_2_ has applications in a variety of processes in the food industry. In breadmaking, 20 to 150 ppm of AH_2_ is commonly dosed to wheat flour for its dough strengthening and bread volume-enhancing effects [5,6,7]. The action of AH_2_ involves oxidation to dihydroascorbic acid (DHA), a process facilitated by AH_2_ oxidases and metal ions, with O_2_ serving as substrate [8]:

2AH_2_ + O_2_ ↔ 2DHA + 2H_2_O (Reaction I)

AH_2_ oxidation is critical in breadmaking, where it promotes disulfide bond formation between gluten proteins. This in turn strengthens the gluten network, which improves its ability to trap fermentation gases and contributes to bread quality. During breadmaking, the oxidation process is predominantly catalyzed enzymatically, with ascorbic acid oxidase delivering about 75% of the DHA production [9,10]. The level of AH_2_ oxidase activity in flour is known to vary, depending on factors such as wheat genotype, agronomic conditions, post-harvest storage, and flour extraction rate [8,9,10,11]. Non-enzymatic oxidation of AH_2_ also occurs, not only in dough, but also in aqueous solutions. This process is mediated primarily by Cu^2+^ and Fe^3+^ metal ions [8,12], either acting directly as a catalyst or serving as co-factor for AH_2_ oxidase (e.g., Cu^2+^) [13]. The non-enzymatic oxidation of AH_2_ involves transition metal ions, the formation of O_2_^−•^, and monodehydroascorbic acid. While the latter undergoes disproportionation to AH_2_ and DHA, O_2_^−•^ generates thiyl radicals from glutathione (GSH) or other thiol-containing molecules. This may participate in reshuffling disulfide bonds within glutenin molecules [14]. Regardless of the oxidation pathway, the availability of dissolved O_2_ governs the production and activity of DHA in the dough matrix, underscoring the importance of AH_2_ oxidation for bread quality.

**Figure 1:**
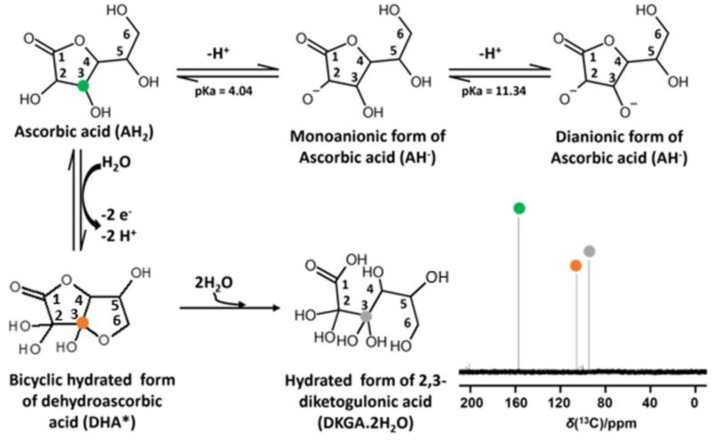
Acid–base reactions of ascorbic acid (AH_2_) and its mono-anionic (AH^−^) and di-anionic (A^2−^) forms and the redox reaction of AH_2_ leading to bicyclic hydrated dehydroascorbic acid (DHA*), which is further hydrolyzed into the hydrated form of 2,3-diketogulonic acid (DKGA.2H_2_O). An example NMR spectrum of AH_2_ dissolved in lab water type-1 after 120 h, indicating the presence of AH_2_, DHA* and DKGA.2H_2_O.

Aside from interacting with the gluten network, DHA can degrade under certain conditions. By prolonged exposure to an oxidative environment or by interactions with other reactive species, DHA can convert into less active or inactive compounds. This occurs by initial conversion to 2,3-diketogulonic acid (DKGA), which further decomposes into gluonic acid, threonic acid, oxalic acid, etc. (Figure 1). DHA can, however, also be regenerated into AH_2_ by glutathione (GSH), endogenously occurring in wheat flour in a range from 18 to 144 nmol GSH/g [11,15]. As GSH reacts with DHA, the latter is reduced to AH_2_, while GSH is dimerized (GSSG) by formation of a disulfide bridge.

DHA + 2GSH → AH_2_ + GSSG (Reaction II)

While AH_2_ oxidation in bread dough can be catalyzed both by enzymes and metal ions, DHA reduction by Reaction I almost exclusively occurs enzymatically [12,16]. Controlled regeneration of DHA by reaction with GSH, a reaction which is facilitated by endogenous GSH dehydrogenase, can therefore assist in protecting AH_2_ against over-oxidation. This potentially mitigates the effects of DHA degradation. An excess of GSH relative to DHA can, however, disrupt the positive effect of DHA on the gluten network by reducing the DHA availability. The interplay between enzymatic and non-enzymatic oxidation, DHA degradation, and GSH-mediated DHA regeneration underscores the complexity of the ascorbic acid chemistry occurring in bread dough. Targeting optimal bread quality, the delicate balance between AH_2_ oxidation and DHA regeneration and degradation mechanisms highlights the need for in situ and ex situ studies investigating the fate of AH_2_ in dough and dough components (e.g., dough liquor) in various conditions, to ultimately achieve precise control of the AH_2_/DHA cycle.

**Scheme A1:**
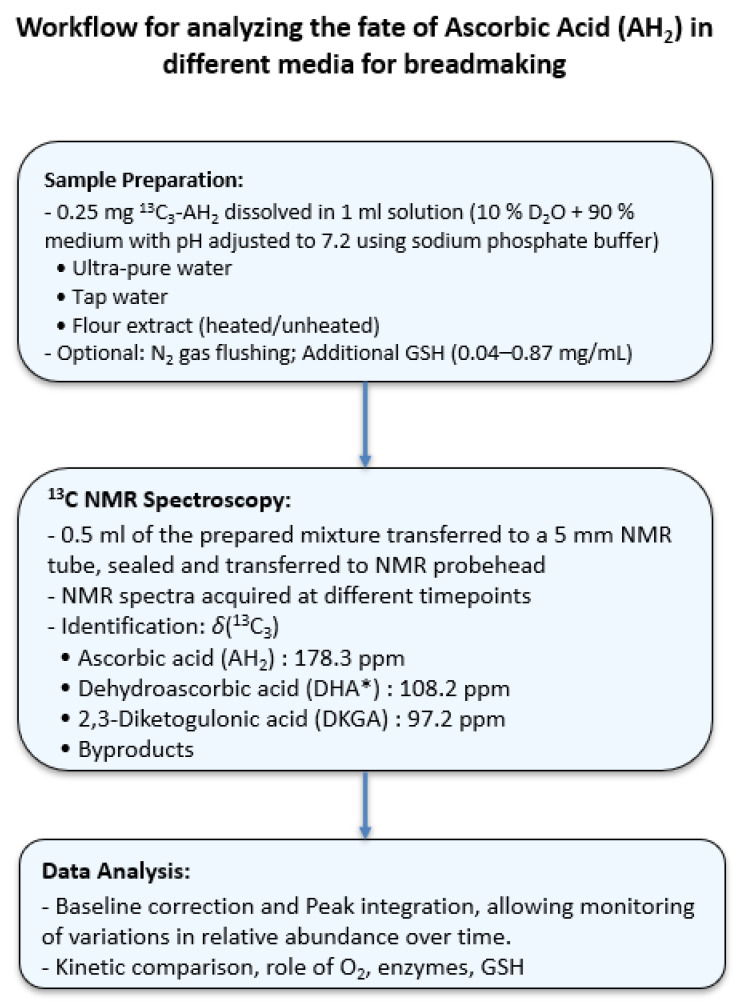
Workflow employed in this study for characterizing and quantifying the transformations of ascorbic acid in different media.

Analysis of AH_2_ and DHA in complex systems is challenging, because of their limited stability. In particular, the spontaneous oxidation of AH_2_ and hydrolysis of DHA are known to be affected by a series of factors including temperature, light, pH, amount of dissolved O_2_, ionic strength of the solvent, and the presence of oxidizing enzymes or divalent cations such as Cu^2+^. This causes problems in the determination of AH_2_ and DHA concentrations [17]. To address these challenges, it is critical to investigate the oxidation–reduction dynamics of AH_2_ in in situ conditions that mimic actual dough matrices with non-destructive techniques providing identification and quantification of the individual species involved. While previous studies had proposed roles for DHA and GSH in dough strengthening, the molecular-level kinetics and transformation pathways of AH_2_ and its oxidation products in aqueous flour systems remain poorly quantified. It is, furthermore, unclear whether endogenous components such as GSH primarily act by directly reducing DHA or by modulating the oxidative environment indirectly; for example, through O_2_ scavenging. The present study employed high-field ^13^C nuclear magnetic resonance (NMR) spectroscopy to monitor the chemical and enzymatic interconversion of AH_2_ and DHA in dough liquor and in synthetic aqueous systems under various conditions—including ultra-pure water, tap water, and pristine and enzyme-deactivated flour extracts (unheated and heated). Adding GSH, metal ions, and modulating the dissolved oxygen concentration assisted in elucidating the respective contributions of enzymatic and non-enzymatic processed. Starting with AH_2,_ labelled with ^13^C on the C_3_ position (^13^C_3_ AH_2_) (Table A1 and Scheme A1), ^13^C NMR spectroscopy allows us to simultaneously determine DHA and AH_2_ concentrations, as well as the concentrations of the DHA degradation products. The approach revealed distinct kinetics for the enzymatic and non-enzymatic pathways, shedding light on the possible regeneration of AH_2_ from DHA* during wheat breadmaking.

## 2. Results and Discussion

### 2.1. Identification of Ascorbic Acid and Its Reaction Products in Water at Ambient Conditions

**Figure A1:**
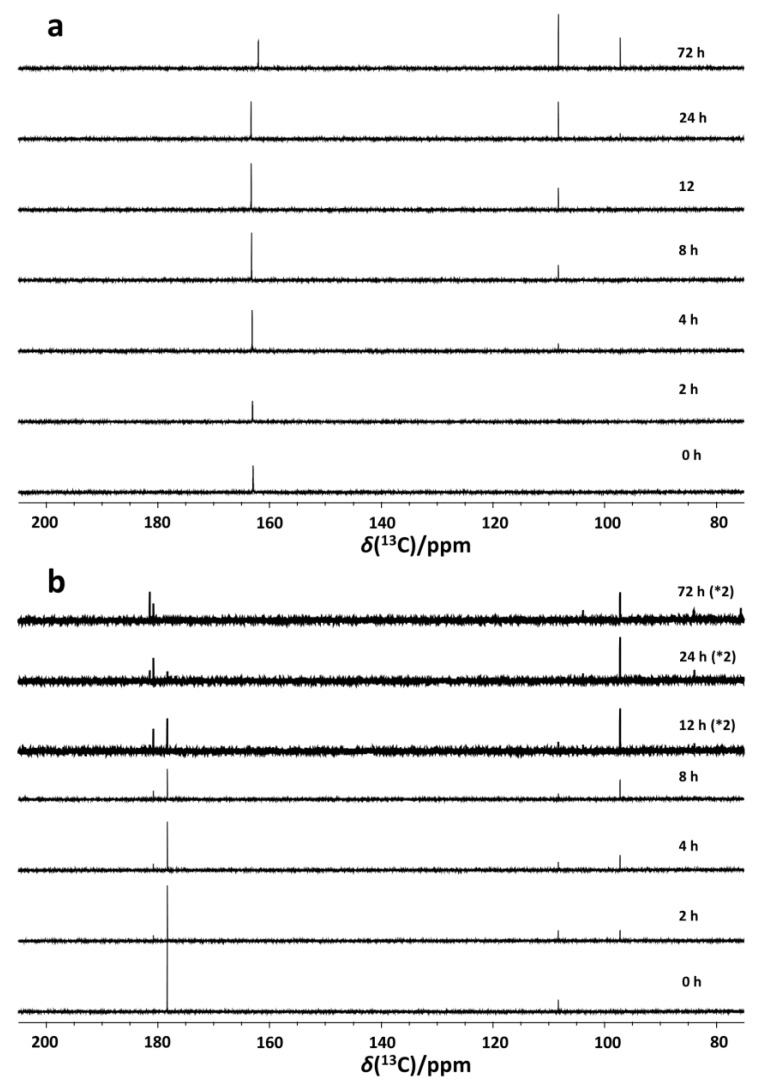
Time evolution of ^13^C NMR spectra of ^13^C_3_-labelled ascorbic acid (^13^C_3_-AH_2_) dissolved in 10% D_2_O containing ultra-pure water (**a**) or tap water (**b**) adjusted to pH 7.2. Chemical shifts of ^13^C_3_-atom of AH_2_ are 162.9 ppm (ultra-pure water) or 178.3 ppm (tap water); chemical shifts of ^13^C_3_-atoms of bicyclic hydrated dehydroascorbic acid (DHA*) and 2,3-diketogulonic acid (DKGA) in the media are 108.2 ppm and 97.2 ppm, respectively.

In ultra-pure water (MilliQ), the chemical shift of the C_3_-atom of freshly dissolved AH_2_ was 162.9 ppm (Appendix A Figure A1a). This is higher than the values reported in the literature, ranging from 155 to 157 ppm [18,19,20]. Dissolved in tap water, the chemical shift of the C_3_ carbon of freshly dissolved AH_2_ was 178.3 ppm (Appendix A Figure A1b). Chemical shifts for the C_3_-atom of AH_2_ solutions derived from Na, Zn or Cd ascorbate salts were previously reported as 176.34, 174.37 and 174.15 ppm, respectively [19]. The difference observed between the ultra-pure- and tap water-based solutions was attributed to the presence of metal ions (Appendix A Table A1) in tap water. Since the other carbon atoms of AH_2_ were not labeled with ^13^C, their resonances remained below the detection limit.

**Figure 2:**
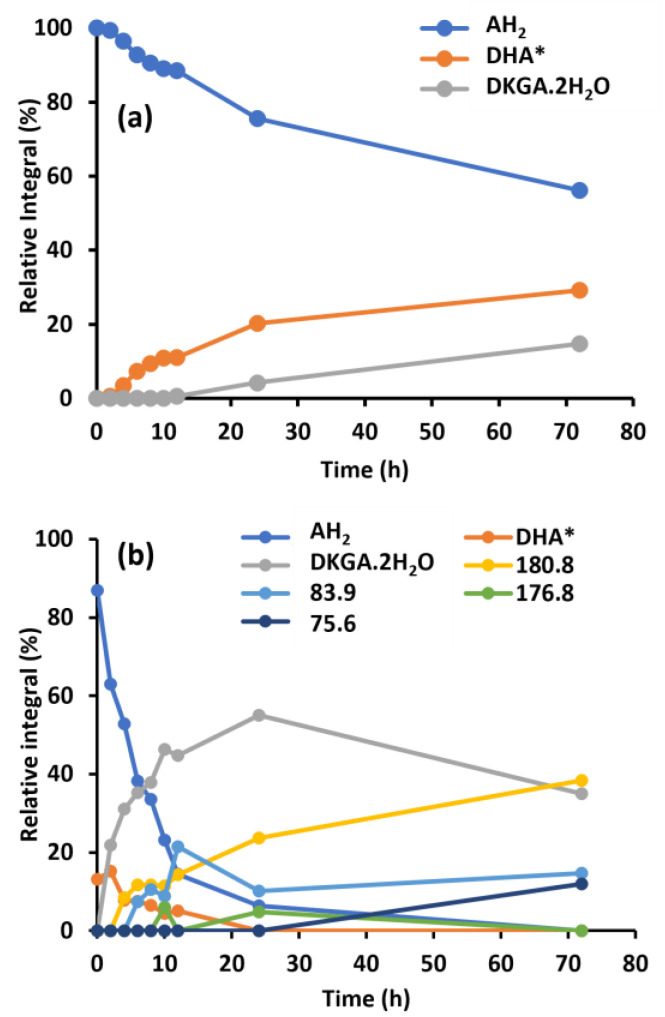
Relative abundance as a function of time of ascorbic acid (AH_2_), bicyclic hydrated dehydroascorbic acid (DHA*), and 2,3-diketogulonic acid (DKGA), as well as of unidentified components estimated from the abundance of their ^13^C signals following dissolution of ^13^C_3_-AH_2_ in ultra-pure water (**a**) or tap water (**b**). Chemical shifts of ^13^C_3_-atom of AH_2_ are 162.9 ppm (pure water) or 178.3 ppm (tap water); chemical shifts of ^13^C_3_-atoms of DHA* and DKGA in the cited media are 108.2 ppm and 97.2 ppm, respectively. ^13^C chemical shifts of the unknown constituents are 180.8, 83.9 and 176.8 ppm.

The transformation of ascorbic acid (AH_2_) either dissolved in tap water or in ultra-pure water is depicted as function of time in Figure 2. In the absence of metal ions (lab water type 1; Figure 2a), DHA* (108.3 ppm) was first detected after four hours. After 24 h, an additional resonance at 97.2 ppm (attributed to DKGA.2H_2_O) was observed. In the tap water-based sample (Figure 2b), oxidation of AH_2_ to DHA* and its subsequent transformations occurred much faster. While in the tap water sample, after 2 h, 15.2% of the AH_2_ was already converted into DHA*, in ultra-pure water the conversion after 2 h only amounted to 0.6%. DKGA is formed by irreversible ring-opening of DHA* (Figure 1). Irrespective of whether DHA* and DKGA were present in ultra-pure or in tap water, the chemical shifts of the C_3_-atoms of both components were identical, implying that only the chemical shift of the C_3_ atom of AH_2_ is affected by the presence of metal ions (Appendix A Figure A1). The ^13^C chemical shift observed for the C_3_-atom in DHA* was comparable to what is reported in the literature: 108.8 ppm in H_2_O [21] or 106.3 ppm in D_2_O [22]. For DKGA.2H_2_O, reported values of 94.4 ppm (pH = 7.0) and 97.4 ppm (pH = 7.4, phosphate buffer) have been reported [23,24].

**Scheme A2:**
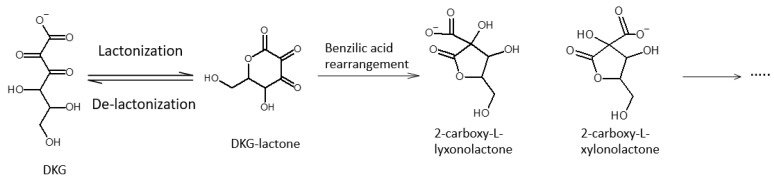
Lactonization of DKG to DKG-lactone, followed by ‘Benzilic acid’ rearrangement, removing one carbon from the six-membered ring to generate a mixture of two epimeric γ-lactones (CPLs: 2-carboxy-L-lyxonolactone and 2-carboxy-L-xylonolactone). These CPLs can undergo further decarboxylation reactions forming xylonates, lyxonates, corresponding lactones, etc. [25].

**Scheme A3:**
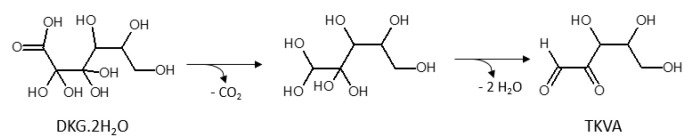
Decarboxylation of hydrated 2,3-diketo-L-gulonic acid (DKG.2H_2_O) to 3,4,5-trihydroxy-2-keto-L-veleraldehyde (TKVA) [24].

After 24 h, 55.0% of the initial AH_2_ concentration was present as DKGA in the tap water sample, as compared to 4.2% in the AH_2_-pure water sample. In the AH_2_-tap water sample, aside from DKGA, degradation products with ^13^C chemical shifts 83.9, 176.8 and 180.8 ppm were also observed from 4 to 6 h onwards. These species could be related to multiple degradation pathways. The resonance at 83.9 and 176.8 ppm may arise due to the formation of 2-carboxy-L-lyxonolactone and 2-carboxy-L-xylonolactone via benzylic acid rearrangement of DKG-lactone (Scheme A2) [25]. Another pathway is the decarboxylation of DKG to 3,4,5-trihydroxy-2-keto-L-valeraldehyde (TKVA) (Scheme A3) [24]. Transition metal ions such as Cu^2+^ and Fe^3+^ have been reported to accelerate AH_2_ oxidation, thus decreasing the stability of AH_2_ [19,26,27,28,29,30]. Both Cu^2+^ and Fe^3+^ are more abundant in tap water than in pure water (Appendix A Table A2). This could explain the different oxidation kinetics observed between both solutions. Simultaneously, AH_2_ transformation kinetics also could be affected by the presence of trace compounds resulting from tap water chlorination [31]. Similar effects have previously also been reported for the oxidation of GSH in tap water versus ultra-pure water. These observations support the validity of the employed NMR methodology using ^13^C-labeled AH_2_ at the C_3_ position for monitoring oxidative transformations. The results also highlight the importance of controlling metal ion content in water to balance AH_2_ oxidation and degradation pathways.

### 2.2. Impact of Molecular Oxygen on the Chemical Conversion of AH_2_

**Figure A2:**
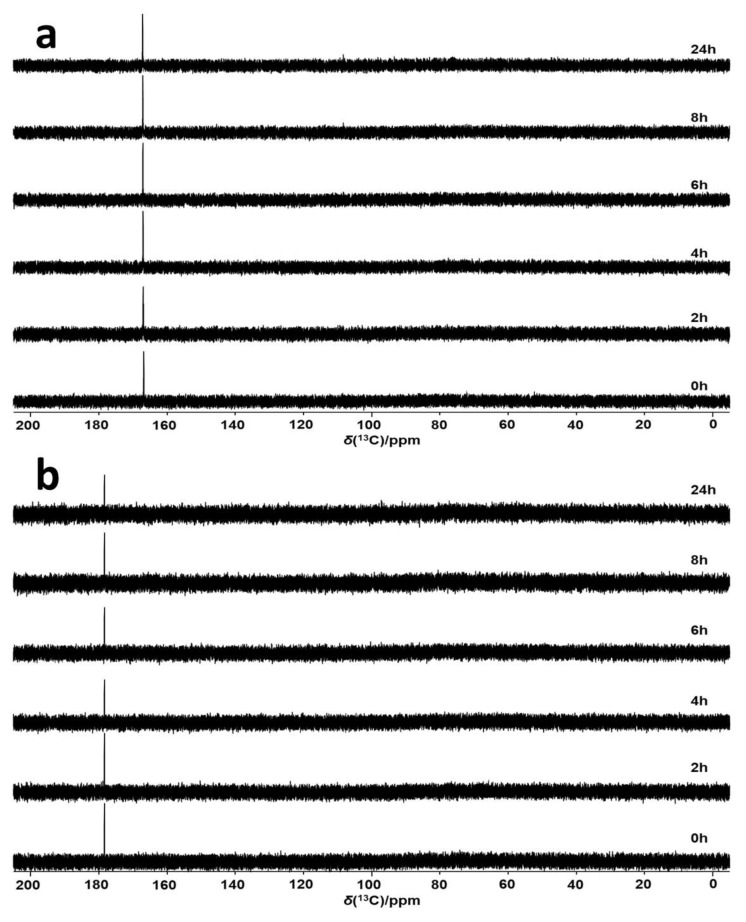
Time evolution of ^13^C NMR spectra of ^13^C_3_-labelled ascorbic acid (^13^C_3_-AH_2_) dissolved in 10% D_2_O containing ultra-pure (**a**) or tap (**b**) water adjusted to pH 7.2 and flushed with nitrogen gas. The chemical shift of the ^13^C_3_-atoms of AH_2_, bicyclic hydrated dehydroascorbic acid (DHA*), and 2,3-diketogulonic acid (DKGA) are 178.3, 108.2 ppm and 97.2 ppm, respectively. The slight intensity decrease in the AH_2_ resonance at 178 ppm in tap water (**b**) is due to increased peak broadening, with no significant additional components detected.

From Reaction I, it can be derived that AH_2_ oxidation should be influenced by the availability of dissolved O_2_ [32]. This was confirmed by flushing ^13^C_3_-AH_2_ solutions with N_2_ gas during preparation, and sealing the samples immediately after. I Both in ultra-pure and in tap water flushed with N_2_, AH_2_ remained much more stable (Figure A2). After 24 h, negligible conversion of AH_2_ was observed in either solution, while only 75.5% and 6.4% of AH_2_, respectively, remained in comparable samples equilibrated with ambient air. These observations confirm that molecular oxygen is a key driver of AH_2_ oxidation, regardless of the aqueous matrix. The pronounced stabilization of AH_2_ under O_2_-lean conditions indicates that oxygen control during dough preparation and conditioning could offer technological opportunities in experimental and industrial settings.

### 2.3. Enzymatic Oxidation of Ascorbic Acid

**Figure 3:**
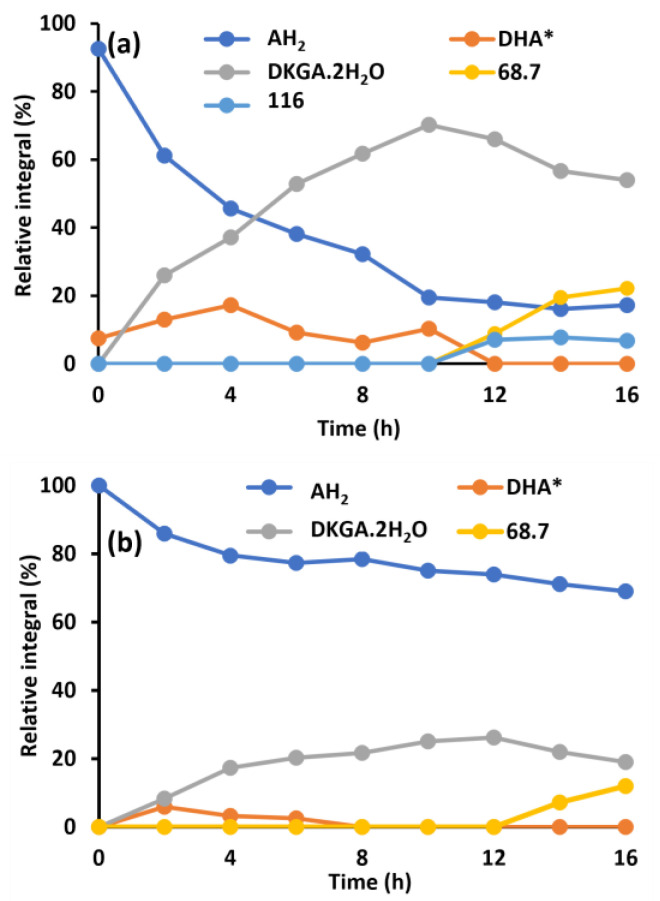
Relative abundance as a function of time of ascorbic acid (AH_2_), bicyclic hydrated dehydroascorbic acid (DHA*), and 2,3-diketogulonic acid (DKGA), as well as of unidentified components estimated from the abundance of their ^13^C signals following dissolution of ^13^C_3_-AH_2_ in aqueous wheat flour extract (**a**) or aqueous heat-treated wheat flour extract (**b**), prepared with tap water. Chemical shifts of ^13^C_3_-atoms of AH_2_, DHA* and DKGA are 178.3 ppm, 108.2 ppm and 97.2 ppm, respectively. ^13^C chemical shifts of the unknown constituents are 116 and 68.7 ppm.

**Figure A3:**
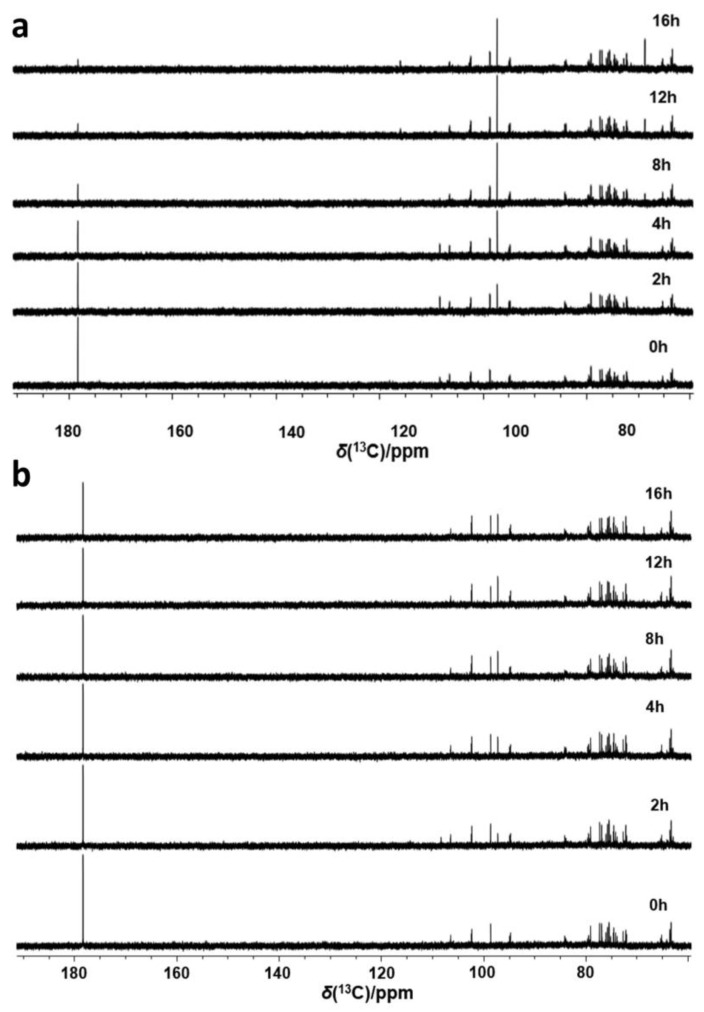
Time evolution of ^13^C NMR spectra of ^13^C_3_-labelled ascorbic acid (^13^C_3_-AH_2_) dissolved in 10% D_2_O containing aqueous unheated (**a**) or heat-treated (**b**) wheat flour extract prepared with tap water and adjusted to pH 7.2. Chemical shifts of ^13^C_3_-atoms of AH_2_, bicyclic hydrated dehydroascorbic acid (DHA*), and 2,3-diketogulonic acid (DKGA) are 178.3 ppm, 108.2 ppm and 97.2 ppm, respectively.

AH_2_ is often used in wheat breadmaking. owing to its dough strengthening and bread volume-enhancing effects [5]. While its bread property-enhancing effects ware well known, the final chemical fate of AH_2_ in breadmaking applications remains nearly undocumented. To compare the enzymatic oxidation of AH_2_ with the chemical oxidation, ^13^C_3_-AH_2_ was dissolved in unheated and in heat-treated aqueous wheat flour extract prepared using tap water. Heat treatment was performed to deactivate the endogenous enzymes. Following its dissolution, the transformation of AH_2_ was monitored over time (Figure 3). The accompanying ^13^C NMR spectra are shown in the Appendix A as Figure A3.

Comparing samples based on untreated (Figure 3a) and heat-treated (Figure 3b) extracts, the heat-induced deactivation of AH_2_ oxidase clearly lowered the rate of AH_2_ oxidation to DHA*. While only 61.1% of AH_2_ remained in the AH_2_-flour extract sample after two hours (Figure 3a), 85.9% AH_2_ was still present in the AH_2_-heated flour extract sample after the same time (Figure 3b). In addition, it was striking that some unidentified components with ^13^C chemical shifts 68.7 and 116 ppm were formed after some time. The latter was formed only in the AH_2_-flour extract sample (Figure 3a), and not in the AH_2_-heated flour extract sample (Figure 3b), nor in the AH_2_-tap water sample (Figure 2b). Figure 3a reveals that once DHA* is formed, it is quickly converted to DKGA. This may indicate that the regeneration of AH_2_ from DHA* by GSH dehydrogenase is far less efficient than that previously assumed by Grosch and Wieser [8]. Peculiarly, after 4 h, the AH_2_ concentration in samples based on heat-treated wheat flour extract (80%) remained significantly higher than that in the plain tap water samples (52.7%). This implies that components in the wheat flour extract, for example endogenous GSH, either prevent AH_2_ oxidation or induce chemical regeneration of DHA* into AH_2_.

### 2.4. The Effect of Glutathione on the Conversion of Ascorbic Acid

In presence of AH_2_ oxidase, GSH, and GSH dehydrogenase, three components endogenously present in wheat flour, AH_2_ could be produced by regeneration of DHA (Reaction IV) [8]. In addition, DHA might also be non-enzymatically reduced to AH_2_ by GSH [33,34]. But GSH is also known to rapidly decrease the available O_2_ concentration in water, as it swiftly oxidizes into GSSG, especially in tap water [35]. The detected AH_2_ in the AH_2_-flour extract sample may thus not only contain initially added, unreacted AH_2_, but also regenerated AH_2_. In addition, overall, the presence of GSH can, therefore, stabilize AH_2_ [36].

To specifically evaluate the impact of these reactions, both AH_2_ and GSH were added to unheated wheat flour extract at concentrations of 0.04, 0.44 or 0.87 mg GSH/mL extract. The two highest GSH concentrations resulted in (1) equimolar levels of GSH and AH_2_ added: GSH = 0.44 mg GSH/mL (1.4 µmol/mL extract); AH_2_ = 0.25 mg/mL (1.4 µmol/mL extract), and (2) a molar ratio of GSH:AH_2_ = 2: GSH: 0.87 mg/mL (2.8 µmol/mL extract); AH_2_: 0.25 mg/mL (1.4 µmol AH_2_/_mL_ extract). The latter was chosen based on Reaction IV, which shows that one DHA molecule (formed from AH_2_) requires two GSH molecules for its regeneration. In neither of these two samples was AH_2_ oxidation observed, even after 16 h.

**Figure 4:**
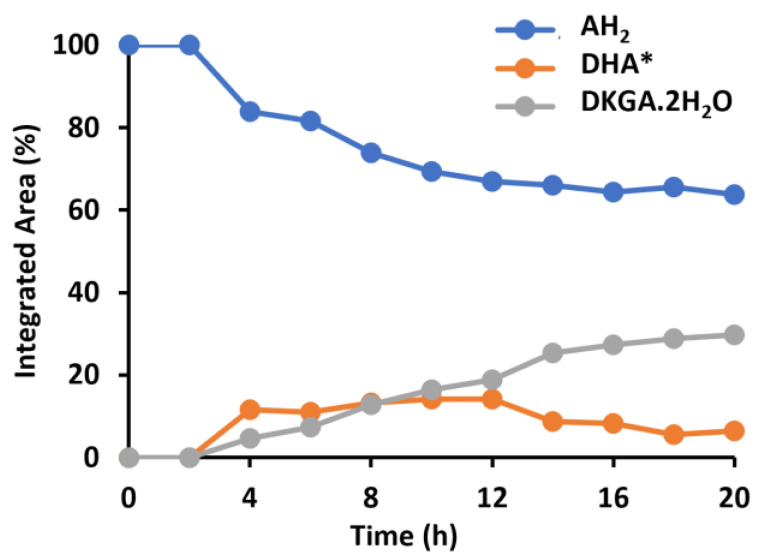
Relative abundance, as a function of time, of ascorbic acid (AH_2_), bicyclic hydrated dehydroascorbic acid (DHA*), and 2,3-diketogulonic acid (DKGA), estimated from the abundance of their ^13^C resonances following dissolution of ^13^C_3_-AH_2_ in aqueous wheat flour extract containing 0.04 mg/mL glutathione (GSH). Chemical shifts of ^13^C_3_-atoms of AH_2_, DHA* and DKGA are 178.3 ppm, 108.2 ppm and 97.2 ppm, respectively.

**Figure A4:**
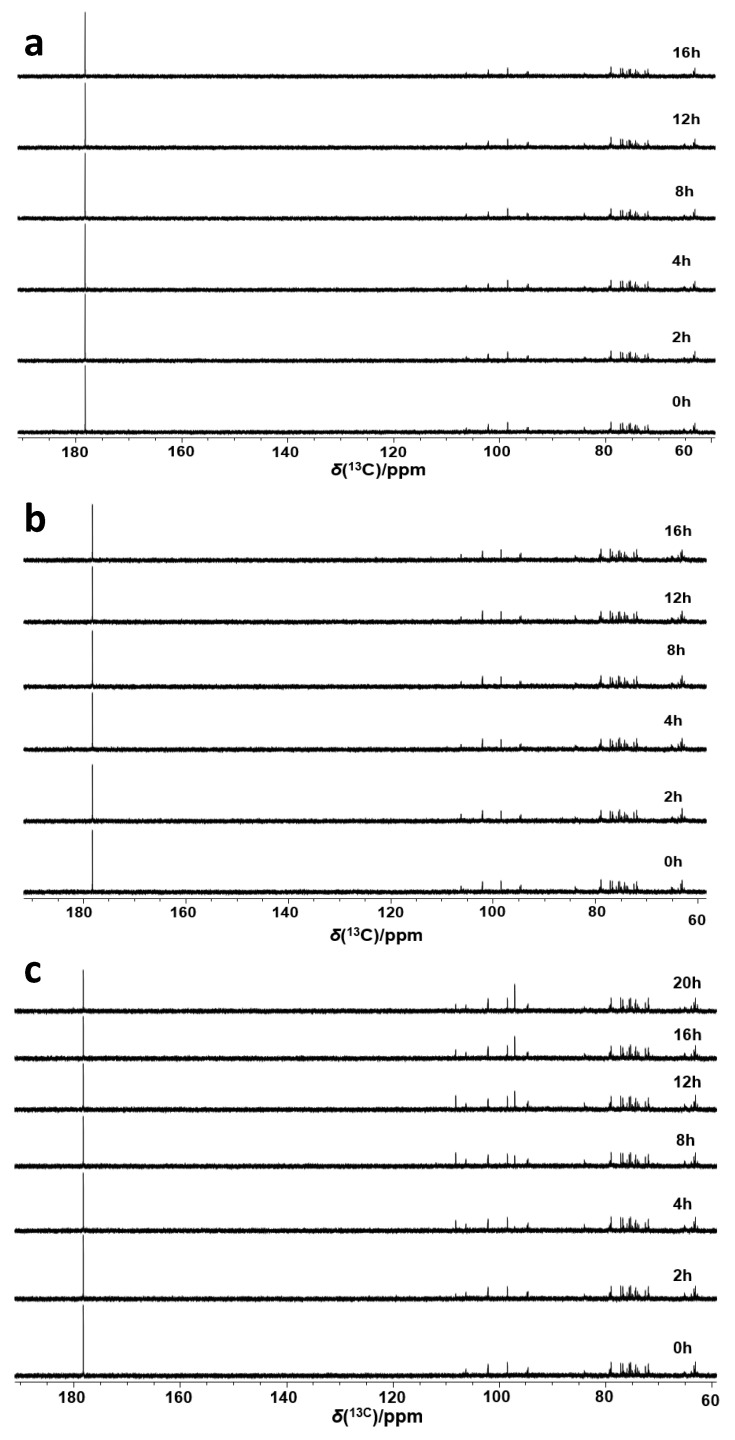
Time evolution of ^13^C NMR spectra of ^13^C_3_-labelled ascorbic acid (^13^C_3_-AH_2_) dissolved in 10% D_2_O containing aqueous unheated wheat flour extract prepared with tap water and adjusted to pH 7.2, to which 0.87 (**a**), 0.44 (**b**), or 0.04 (**c**) mg/mL glutathione (GSH) was added. Chemical shifts of ^13^C_3_-atoms of AH_2_, bicyclic hydrated dehydroascorbic acid (DHA*), and 2,3-diketogulonic acid (DKGA) are 178.3 ppm, 108.2 ppm and 97.2 ppm, respectively.

Figure 4 shows the evolution of the dosed AH_2_ concentration, as a function of time, for a sample with added AH_2_:GSH molar ratio of 10 (0.04 mg GSH/mL extract). The corresponding ^13^C NMR spectra are shown in Appendix A Figure A4. In this last sample, DHA* and DKGA were detected, indicating the oxidation of AH_2_ (Figure 4). Comparing the time evolution of the AH_2_ levels in AH_2_-flour extract samples, with (Figure 4) and without (Figure 3a) added GSH, it was observed that the addition of GSH in a 1/10 GSH/AH_2_ ratio nearly doubled the remaining AH_2_ concentration after 4 h (84% vs. 46%, respectively). In addition, after four hours, only 4.6% DKGA was detected in this sample, compared to 37% in the AH_2_-flour extract sample. When high GSH levels were added, 100% AH_2_ was still present after 16 h (Appendix A Figure A4) compared to only 17.2% in the AH_2_-flour extract sample (Figure 3a). Overall, these results indicate that, rather than serving as electron donor for the regeneration of DHA* to AH_2_, GSH primarily acts on decreasing the availability of dissolved O_2_. This mechanistic insight may inform optimized antioxidant strategies in food formulations. It also highlights the need to consider GSH levels in commercial flour as a variable influencing dough behavior.

## 3. Materials and Methods

### 3.1. Materials and Chemicals

Additive-free wheat flour (Crousti) was provided by Puratos NV (Groot-Bijgaarden, Belgium) [7,37]. Its moisture (14.6%) and protein (13.1% of dry matter) contents were determined in triplicate, according to the AACC method 44-15-02 (1999) and an adaptation of the AOAC method 990.03 (1995) to an automated Dumas protein analysis system (VarioMax Cube N, Elementar, Hanau, Germany), respectively. According to the supplier, the ash content was 0.57% ± 0.03. All chemicals, reagents, and solvents were purchased from Merck Life Science (Overijse, Belgium) and were of analytical grade. Pure water with a resistivity of 18.2 MΩ.cm at 25 °C was obtained using a Millipore Milli-Q lab water system (Merck MilliPore, Burlington, MA, USA).

### 3.2. Preparation of the Different Aqueous Solutions

AH_2_’s oxidation to DHA* and its possible regeneration were studied in media, the codes of which are listed in Table A1. Cations in pure water and tap water were quantified by inductively coupled plasma mass spectrometry, using an Agilent 7700× (Agilent Technologies, Santa Clara, CA, USA), the results of which are given in Appendix A Table A2.

Wheat flour extract was obtained by suspending 10.0 g wheat flour in 20.0 mL tap water, shaking (10 min, 150 rpm), and centrifuging (10 min; 5000× *g*). Wheat flour extracts were heat-reated to inactivate endogenous wheat flour AH_2_ oxidase and GSH dehydrogenase. Following this, the extracts were kept at 70 °C for 30 min and then allowed to cool to room temperature. Pfeilsticker and Roeung observed minimal AH_2_ oxidase activity after heating wheat flour extract for 60 min at 60 °C, and Every et al. reported full inactivation of GSH dehydrogenase when flour extract was heated for 20 min at 70 °C [12,38].

The different media (Table A1) were adjusted to pH 7.2 using 0.1 M sodium phosphate buffer (pH 7.4) prepared with pure water. One volume of D_2_O was added to nine volumes of pure water, tap water, or (heat-treated) wheat flour extract.

Where appropriate (Table A1), O_2_ was eliminated from the NMR tube and different media before and after AH_2_ addition by flushing with dry nitrogen (N_2_) gas. GSH was added to certain wheat flour extract samples (Table A1) after pH adjustment and D_2_O addition, and immediately before ^13^C_3_-AH_2_ was added. To obtain 0.04 mg GSH/mL flour extract, 0.40 mg GSH was first dissolved in 10.0 mL 10% D_2_O containing aqueous flour extract, while 0.44 or 0.87 mg GSH wasdissolved in 1.00 mL 10% D_2_O containing aqueous flour extract.

^13^C_3_-AH_2_ aliquots (0.25 mg) were accurately weighed in Eppendorf tubes. Immediately before the start of the NMR measurements, 1.0 mL of the different 10% D_2_O-containing samples was added to the Eppendorf tube, of which 0.5 mL was transferred to a 5 mm NMR tube, which was then closed with a polyethylene cap.

### 3.3. ^13^C Liquid-State Nuclear Magnetic Resonance Measurements

^1^H decoupled ^13^C NMR spectra were acquired on an 800 MHz Bruker Avance Neo spectrometer (Bruker Belgium NV, Kontich, Belgium) equipped with a 5 mm multinuclear BBO probe (1H/2D/X). ^13^C direct excitation NMR spectra were acquired using an excitation pulse with 30-degree flip angle at a 25 kHz radio frequency, recycle delay of 4 s, and 720 transients. The spectra were referenced to tetramethylsilane, using the secondary reference sodium trimethylsilyl-propanesulfonate in D_2_O at 0.0173 ppm [39]. The experiments were performed by following procedures for absolute quantitative NMR [40,41].

Phase correction, baseline correction, and integration of the ^13^C NMR spectra were performed using the Bruker TopSpin 4.0.9 software. For each time point, the integrals of the C_3_-carbon signals corresponding to AH_2_, DHA*, DKGA, and other identifiable degradation products were quantified. The relative abundance of each component was calculated by normalizing its integrated signal area to the total integrated area of all detected ^13^C signals. This allowed time-resolved tracking of AH_2_ transformation pathways under the investigated experimental conditions.

## 4. Conclusions

Ascorbic acid (AH_2_) is often used in wheat breadmaking. owing to its dough strengthening and bread volume-enhancing effects. While these bread property-enhancing effects are well known, the final chemical fate of AH_2_ in breadmaking applications remains nearly undocumented. This study investigated the chemical fate of AH_2_ in relevant wheat flour extract, evaluating the specific impact of dynamic processes which could be involved in the oxidation of AH_2_. Using various aqueous media, including oxygenated, as well as deoxygenated, tap water and ultra-pure water, the impact of dissolved oxygen, trace metal ions and trace reaction products of chlorine-based water disinfection were evaluated. These observations underscore the validity of the NMR approach employing ^13^C-labeled AH_2_ and the critical importance of monitoring metal ion content in aqueous systems when aiming to balance oxidation and degradation of AH_2_. To discriminate between enzymatic and non-enzymatic processes, the fate of AH_2_ in heat-treated and in pristine wheat flour extract was evaluated as a function of time. Finally, both glutathione (GSH) and AH_2_ were added to pristine wheat flour extracts in molar ratios of GSH:AH_2_ = 2, 1 and 0.1, to elucidate the impact of endogenous GSH on the oxidation and/or regeneration of AH_2_. Remarkably, reducing the dissolved O_2_ concentration in both media by purging with N_2_ gas significantly enhanced the AH_2_ stability, as evidenced by the limited formation of DHA* and hydrolysis to DKGA. While the oxidation of AH_2_ in water was strongly enhanced by the presence of trace metal ions and/or trace reaction products of water disinfection, surprisingly, the impact of these components in solutions based on wheat flour extract was reduced. By introducing different ratios of both GSH and AH_2_ into wheat flour extract, it was shown that the dominant impact of endogenous GSH on AH_2_ availability must be to decrease the availability of dissolved oxygen, rather than to assist in the regeneration of DHA* in AH_2_ by GSH dehydrogenase. This points to the potential for regulating dough redox balance by managing GSH levels and oxygen exposure during mixing and fermentation.. These insights into the stability and transformation of AH_2_ could spark new strategies for optimizing dough conditioning by managing the balance between oxidation and regeneration of ascorbic acid, not only through redox additives, but also by modulating flour composition or by controlling the process atmosphere. Future research is needed to translate these findings to technologically relevant modifications to dough and breadmaking processes. Coupling NMR-based techniques with functional dough assays may help bridge molecular-level transformations with macroscopic bread quality outcomes. In situ monitoring of AH_2_ transformations during dough processing, enabled by the advent of high-pressure MAS rotors [42] capable of containing CO_2_ release, in combination with resolution enhancement techniques [43], represents an exciting scientific avenue.

## Data Availability

Replication data for the NMR related figures in the main manuscript is available via Harvard Dataverse https://doi.org/10.7910/DVN/HRHKPD. All the relevant NMR spectra are provided in the Appendix A to the manuscript.

## Appendix A

**Table A1:** Overview of the samples and the media used.

Sample	Solvent Used	Prepared Under Atmospheric Conditions or Flushed with N_2_
AH_2_ in Ultra-pure water	Ultra-pure water (MilliQ, Millipore)	Atmospheric conditions
AH_2_ in Tap water	Tap water	Atmospheric conditions
AH_2_ in Ultra-pure water—N_2_ treated	Ultra-pure water (MilliQ, Millipore)	Flushed with N_2_
AH_2_ in Tap water—N_2_ treated	Tap water	Flushed with N_2_
AH_2_ in Flour extract	^1^ Flour extract in tap water	Atmospheric conditions
AH_2_ in Heated flour extract	^1^ Heat-treated (70 °C—30 min) flour extract	Atmospheric conditions
AH_2_ in Flour extract—N_2_ treated	^1^ Flour extract in tap water	Flushed with N_2_
AH_2_-Flour + 0.04 GSH-Atm	0.04 mg/mL GSH added to the flour extract	Atmospheric conditions
AH_2_-Flour + 0.44 GSH-Atm	0.44 mg/mL GSH added to the flour extract	Atmospheric conditions
AH_2_-Flour + 0.87 GSH-Atm	0.87 mg/mL GSH added to the flour extract	Atmospheric conditions

^1^ Wheat flour extract was obtained by suspending 10.0 g wheat flour in 20.0 mL tap water, shaking (10 min, 150 rpm), and centrifuging (10 min; 5000 g). Wheat flour extracts were heat-treated to inactivate endogenous wheat flour AH_2_ oxidase and GSH dehydrogenase. Following this, the extracts were kept at 70 °C for 30 min and then allowed to cool to room temperature.

**Table A2:** Concentrations (µg/L) of the different transition metal ions in pure and tap water determined by inductively coupled plasma mass spectrometry.

Transition Metal Ion	Concentration (µg/L)	
	Pure Water	Tap Water
Zn	5.71	89.76
Fe	2.91	50.88
Cu	0.30	23.54
Cd	0.30	1.17
Ni	0.14	1.20
Mn	0.05	0.78
Mo	0.03	0.96
Co	0.01	−0.01

## References

[1] Ali A., Riaz S., Khalid W., Fatima M., Mubeen U., Babar Q., Manzoor M.F., Zubair Khalid M., Madilo F.K. (2024). Potential of Ascorbic Acid in Human Health against Different Diseases: An Updated Narrative Review. Int. J. Food Prop..

[2] Mittu B., Bhat Z.R., Chauhan A., Kour J., Behera A., Kaur M. (2021). Ascorbic Acid.

[3] Naidu K.A. (2013). Vitamin C in Human Health and Disease Is Still a Mystery? An Overview. Funct. Foods Connect. Nutr. Health Food Sci..

[4] Padayatty S.J., Levine M. (2016). Vitamin C: The Known and the Unknown and Goldilocks. Oral Dis..

[5] Joye I.J., Lagrain B., Delcour J.A. (2009). Use of Chemical Redox Agents and Exogenous Enzymes to Modify the Protein Network during Breadmaking–A Review. J. Cereal Sci..

[6] Koehler P. (2003). Effect of Ascorbic Acid in Dough:  Reaction of Oxidized Glutathione with Reactive Thiol Groups of Wheat Glutelin. J. Agric. Food Chem..

[7] Beghin A.S., Ooms N., Brijs K., Pareyt B., Moldenaers P., Delcour J.A. (2021). How Yeast Impacts the Effect of Ascorbic Acid on Wheat Flour Dough Extensional Rheology. Food Biophys..

[8] Grosch W., Wieser H. (1999). Redox Reactions in Wheat Dough as Affected by Ascorbic Acid. J. Cereal Sci..

[9] Every D., Gilpin M.J., Larsen N.G. (1995). Continuous Spectrophotometric Assay and Properties of Ascorbic Acid Oxidising Factors in Wheat. J. Cereal Sci..

[10] Every D., Simmons L.D., Ross M.P. (2006). Distribution of Redox Enzymes in Millstreams and Relationships to Chemical and Baking Properties of Flour. Cereal Chem..

[11] Schofield J.D., Chen X. (1995). Analysis of Free Reduced and Free Oxidised Glutathione in Wheat Flour. J. Cereal Sci..

[12] Every D., Simmons L., Sutton K.H., Ross M. (1999). Studies on the Mechanism of the Ascorbic Acid Improver Effect on Bread Using Flour Fractionation and Reconstitution Methods. J. Cereal Sci..

[13] Kuninori T., Matsumoto H. (1963). L-Ascorbic Acid Oxidizing System in Dough and Dough Improvement. Cereal Chem..

[14] Nakamura M., Kurata T. (1997). Effect of L-Ascorbic Acid on the Rheological Properties of Wheat Flour-Water Dough. Cereal Chem..

[15] Li W., Bollecker S.S., Schofield J.D. (2004). Glutathione and Related Thiol Compounds. I. Glutathione and Related Thiol Compounds in Flour. J. Cereal Sci..

[16] Dong W., Hoseney R.C. (1995). Effects of Certain Breadmaking Oxidants and Reducing Agents on Dough Rheological Properties. Cereal Chem..

[17] Washko P.W., Welch R.W., Dhariwal K.R., Wang Y., Levine M. (1992). Ascorbic Acid and Dehydroascorbic Acid Analyses in Biological Samples. Anal. Biochem..

[18] Paukstelis J.V., Mueller D.D., Seib P.A., Lillard D.W. (1982). NMR Spectroscopy of Ascorbic Acid and Its Derivatives. Ascorbic Acid: Chemistry, Metabolism, and Uses.

[19] Tajmir-Riahi H.A. (1991). Coordination Chemistry of Vitamin C. Part II. Interaction of L-Ascorbic Acid with Zn(II), Cd(II), Hg(II), and Mn(II) Ions in the Solid State and in Aqueous Solution. J. Inorg. Biochem..

[20] Albertino A., Barge A., Cravotto G., Genzini L., Gobetto R., Vincenti M. (2009). Natural Origin of Ascorbic Acid: Validation by 13C NMR and IRMS. Food Chem..

[21] Kerber R.C. (2008). “As Simple as Possible, but Not Simpler”—The Case of Dehydroascorbic Acid. J. Chem. Educ..

[22] Hvoslef J., Pedersen B., Wennerström O., Enzell C.R., Åkeson Å., Lundquist G. (1979). The Structure of Dehydroascorbic Acid in Solution. Acta Chem. Scand..

[23] Tolbert B.M., Ward J.B. (1982). Dehydroascorbic Acid. Ascorbic Acid Chem. Metab. Uses.

[24] Kang S.-O., Sapper H., Lohmann W. (1982). The Oxidative Degradation of ʟ-Ascorbic Acid via an α-Ketoaldehyde. Zeitschrift für Naturforsch. C.

[25] Dewhirst R.A., Murray L., Mackay C.L., Sadler I.H., Fry S.C. (2020). Characterisation of the Non-Oxidative Degradation Pathway of Dehydroascorbic Acid in Slightly Acidic Aqueous Solution. Arch. Biochem. Biophys..

[26] Buettner G.R., Jurkiewicz B.A. (1996). Catalytic Metals, Ascorbate and Free Radicals: Combinations to Avoid. Radiat. Res..

[27] Khan M.M.T., Martell A.E. (1967). Metal Ion and Metal Chelate Catalyzed Oxidation of Ascorbic Acid by Molecular Oxygen. I. Cupric and Ferric Ion Catalyzed Oxidation. J. Am. Chem. Soc..

[28] Liao M.L., Seib P.A. (1988). Chemistry of L-Ascorbic Acid Related to Foods. Food Chem..

[29] Moya H.D., Coichev N. (2006). Kinetic Studies of the Oxidation of L-Ascorbic Acid by Tris(Oxalate)Cobaltate in the Presence of CDTA Metal Ion Complexes. J. Braz. Chem. Soc..

[30] Jansson P.J., Jung H.R., Lindqvist C., Nordström T. (2004). Oxidative Decomposition of Vitamin C in Drinking Water. Free Radic. Res..

[31] Legay C., Rodriguez M.J., Sérodes J.B., Levallois P. (2010). Estimation of Chlorination By-Products Presence in Drinking Water in Epidemiological Studies on Adverse Reproductive Outcomes: A Review. Sci. Total Environ..

[32] Chwastowski J., Ciesielski W., Khachatryan K., Kołoczek H., Kulawik D., Oszczęda Z., Soroka J.A., Tomasik P., Witczak M. (2020). Water of Increased Content of Molecular Oxygen. Water.

[33] Sapper H., Paul H.-H., Lohmann W., Institut für Biophysik der Justus-Liebig-Universität Gießen Sa-Ouk (1982). The Reversibility of the Vitamin C Redox System: Electrochemical Reasons and Biological Aspects. Zeitschrift für Naturforschung C.

[34] Winkler B.S., Orselli S.M., Rex T.S. (1994). The Redox Couple between Glutathione and Ascorbic Acid: A Chemical and Physiological Perspective. Free Radic. Biol. Med..

[35] Lauwers K., Breynaert E., Rombouts I., Delcour J.A., Kirschhock C.E.A. (2016). Water Electrolyte Promoted Oxidation of Functional Thiol Groups. Food Chem..

[36] Touitou E., Alkabes M., Memoli A., Alhaique F. (1996). Glutathione Stabilizes Ascorbic Acid in Aqueous Solution. Int. J. Pharm..

[37] Beghin A.S., Ooms N., Brijs K., Pareyt B., Delcour J.A. (2022). Release of 14C-Labeled Carbon Dioxide from Ascorbic Acid during Straight Dough Wheat Bread Making. Cereal Chem..

[38] Pfeilsticker K., Roeung S. (1982). Charakterisierung Derl-Ascorbinsureoxidase (EC 1.10.3.3.) Aus Weizenmehl. Z. Fur Lebensm. Und-Forschungr Lebensm. Und-Forsch..

[39] Harris R.K., Becker E.D., Cabral de Menezes S.M., Goodfellow R., Granger P. (2001). NMR Nomenclature. Nuclear Spin Properties and Conventions for Chemical Shifts(IUPAC Recommendations 2001). Pure Appl. Chem..

[40] Houlleberghs M., Hoffmann A., Dom D., Kirschhock C.E.A., Taulelle F., Martens J.A., Breynaert E. (2017). Absolute Quantification of Water in Microporous Solids with 1 H Magic Angle Spinning NMR and Standard Addition. Anal. Chem..

[41] Vanderschaeghe H., Houlleberghs M., Verheyden L., Dom D., Chandran C.V., Radhakrishnan S., Martens J.A., Breynaert E. (2022). Absolute Quantification of Residual Solvent in Mesoporous Silica Drug Formulations Using Magic-Angle Spinning NMR Spectroscopy. Anal. Chem..

[42] Chamas A., Qi L., Mehta H.S., Sears J.A., Scott S.L., Walter E.D., Hoyt D.W. (2019). High Temperature/Pressure MAS-NMR for the Study of Dynamic Processes in Mixed Phase Systems. Magn. Reson. Imaging.

[43] De Man W.L., Chandran C.V., Wouters A.G.B., Radhakrishnan S., Martens J.A., Breynaert E., Delcour J.A. (2022). Hydration of Wheat Flour Water-Unextractable Cell Wall Material Enables Structural Analysis of Its Arabinoxylan by High-Resolution Solid-State 13C MAS NMR Spectroscopy. J. Agric. Food Chem..

